# A One Health Comparative Assessment of Antimicrobial Resistance in Generic and Extended-Spectrum Cephalosporin-Resistant *Escherichia coli* from Beef Production, Sewage and Clinical Settings

**DOI:** 10.3390/microorganisms8060885

**Published:** 2020-06-11

**Authors:** Emelia H. Adator, Claudia Narvaez-Bravo, Rahat Zaheer, Shaun R. Cook, Lisa Tymensen, Sherry J. Hannon, Calvin W. Booker, Deirdre Church, Ron R. Read, Tim A. McAllister

**Affiliations:** 1 Department of Food and Human Nutritional Sciences, University of Manitoba, Winnipeg, MB R3T 2N2, Canada; adatore@myumanitoba.ca (E.H.A.); Claudia.NarvaezBravo@umanitoba.ca (C.N.-B.); 2 Lethbridge Research and Development Centre, Agriculture and Agri-Food Canada, Lethbridge, AB T1J 4B1, Canada; rahat.zaheer@canada.ca; 3 Irrigation and Farm Water Branch, Alberta Agriculture and Forestry, Lethbridge, AB T1J 4V6, Canada; Shaun.Cook@gov.ab.ca (S.R.C.); lisa.tymensen@gov.ab.ca (L.T.); 4 Health Management Services Ltd, Okotoks, AB T1S 2A2, Canada; sherryh@feedlothealth.com (S.J.H.); calvinb@feedlothealth.com (C.W.B.); 5 Department of Pathology & Laboratory Medicine and Medicine, Cumming School of Medicine, University of Calgary, Calgary, AB T2N 4N1, Canada; deirdre.church@cls.ab.ca (D.C.); rread1@gmail.com (R.R.R.)

**Keywords:** antimicrobial resistance, extended-spectrum beta-lactamase (ESBL), one health, beef, sewage

## Abstract

This study aimed to compare antimicrobial resistance (AMR) in extended-spectrum cephalosporin-resistant and generic *Escherichia coli* from a One Health continuum of the beef production system in Alberta, Canada. A total of 705 extended-spectrum cephalosporin-resistant *E. coli* (ESC^r^) were obtained from: cattle feces (CFeces, *n* = 382), catch basins (CBasins, *n* = 137), surrounding streams (SStreams, *n* = 59), beef processing plants (BProcessing, *n* = 4), municipal sewage (MSewage; *n* = 98) and human clinical specimens (CHumans, *n* = 25). Generic isolates (663) included: CFeces (*n* = 142), CBasins (*n* = 185), SStreams (*n* = 81), BProcessing (*n* = 159) and MSewage (*n* = 96). All isolates were screened for antimicrobial susceptibility to 9 antimicrobials and two clavulanic acid combinations. In ESC^r^*,* oxytetracycline (87.7%), ampicillin (84.4%) and streptomycin (73.8%) resistance phenotypes were the most common, with source influencing AMR prevalence (*p* < 0.001). In generic *E. coli,* oxytetracycline (51.1%), streptomycin (22.6%), ampicillin (22.5%) and sulfisoxazole (14.3%) resistance were most common. Overall, 88.8% of ESC^r^, and 26.7% of generic isolates exhibited multi-drug resistance (MDR). MDR in ESC^r^ was high from all sources: CFeces (97.1%), MSewage (96.9%), CHumans (96%), BProcessing (100%), CBasins (70.5%) and SStreams (61.4%). MDR in generic *E. coli* was lower with CFeces (45.1%), CBasins (34.6%), SStreams (23.5%), MSewage (13.6%) and BProcessing (10.7%). ESBL phenotypes were confirmed in 24.7% (*n* = 174) ESC^r^ and 0.6% of generic *E. coli*. Prevalence of *bla* genes in ESC^r^ were *bla*_CTXM_ (30.1%), *bla*_CTXM-1_ (21.6%), *bla*_TEM_ (20%), *bla*_CTXM-9_ (7.9%), *bla*_OXA_ (3.0%), *bla*_CTXM-2_ (6.4%), *bla*_SHV_ (1.4%) and AmpC β-lactamase *bla*_CMY_ (81.3%). The lower AMR in ESC^r^ from SStreams and BProcessing and higher AMR in CHumans and CFeces likely reflects antimicrobial use in these environments. Although MDR levels were higher in ESC^r^ as compared to generic *E. coli*, AMR to the same antimicrobials ranked high in both ESC^r^ and generic *E. coli* sub-populations. This suggests that both sub-populations reflect similar AMR trends and are equally useful for AMR surveillance. Considering that MDR ESC^r^ MSewage isolates were obtained without enrichment, while those from CFeces were obtained with enrichment, MSewage may serve as a hot spot for MDR emergence and dissemination.

## 1. Introduction

Antimicrobial resistance (AMR) is a critical public health risk, estimated to account for 700,000 human mortalities per year [1,2]. Drivers of AMR include profuse use of antimicrobials in human and veterinary medicine, often misused in clinical settings or overused to improve the efficiency of livestock and crop production [3]. Genes associated with AMR can circulate among bacteria which may contaminate food or water consumed by humans [3,4,5].

Canada is ranked among the top 10 beef exporters worldwide. In 2016, Canada’s beef export industry was valued at $2.3 billion, contributing $33 billion worth of goods and services to the nation [6]. Antimicrobials (i.e., antibiotics) are administered to cattle to prevent and treat various diseases such as liver abscesses [7], foot rot [8] and bovine respiratory diseases (BRD) [9,10]. Owing to the routine use of antimicrobials common to the same class in both humans and beef, there is a possibility of antimicrobial use (AMU) in beef contributing to AMR in humans. Some studies have found similar AMR profiles and prevalence in bacterial species such as *Escherichia coli* and *Salmonella* isolates and AMR genes from cattle, humans, swine and waste streams [11,12,13]. Several studies have been narrower in scope, focusing on farms [14], processing plants [15,16] and retail meat [17]. Although a One Health approach to AMR monitoring has been advocated, most studies have not systematically compared AMR *E. coli* isolated across the beef production chain to those obtained from human sources. The One Health approach acknowledges the interconnectedness of health domains associated with humans, animals and the environment, and employs an integrated approach to risk management and decision making. This enables the identification of those segments along the continuum that can best disrupt the transmission of AMR from the environment to humans [2,18].

Extended-spectrum β-lactam (ESBL) resistant *E. coli* are of particular public health concern [19] as they are able to inactivate most β-lactam antimicrobials [20] used to treat associated infections. Presently, ESBL bacteria are a common source of therapeutic failure due to frequent co-resistance to multiple last resort antimicrobials [21,22] including colistin (i.e., polymyxin E), aminoglycoside, and 3rd and 4th generation cephalosporins that are used to treat a significant proportion of nosocomial infections [23,24,25]. In veterinary and human medicine, the extended-spectrum cephalosporins which are a sub-class of ESBL are regarded as critically important [26]. Resistance in extended-spectrum cephalosporin-resistant *E. coli* is often encoded by genes such as *bla*_SHV_, *bla*_TEM_ and *bla*_CTX-M_ [22,27].

Monitoring of cephalosporin-resistant bacteria in agricultural sectors could uncover crucial information for designing cost-effective actions to minimize the disease burden associated with these bacterial infections [28]. Considering the impact of such infections on human health, broadening the scope of monitoring to include AmpC-producing bacteria has also been recommended by the WHO [18]. A study by Horton et al. [29] found levels of CTX-M-positive *E. coli* in cattle, pigs and chickens at 1002, 800 and 5350 CFU/g of feces, respectively. Even though cephalosporin-resistant *E. coli* have been reported in cattle, cattle farms and retail beef [30,31], evidence supporting the direct contribution of livestock production to AMR emergence along the One Health continuum is lacking. In contrast to the low prevalence of extended-spectrum cephalosporin-resistant *E. coli* (ESC^r^) reported by the Canadian Integrated Program for Antimicrobial Resistance, Cormier et al. recently [32] found a high prevalence of ESC^r^ in cattle using enrichment methods. The present study aimed to employ a One Health approach to examine similarities between patterns and prevalence of AMR typical of the entire food continuum using generic *E. coli* and cephalosporin-resistant *E. coli* in a defined geographic region of high beef production intensity in Canada.

## 2. Materials and Methods

**Figure 1:**
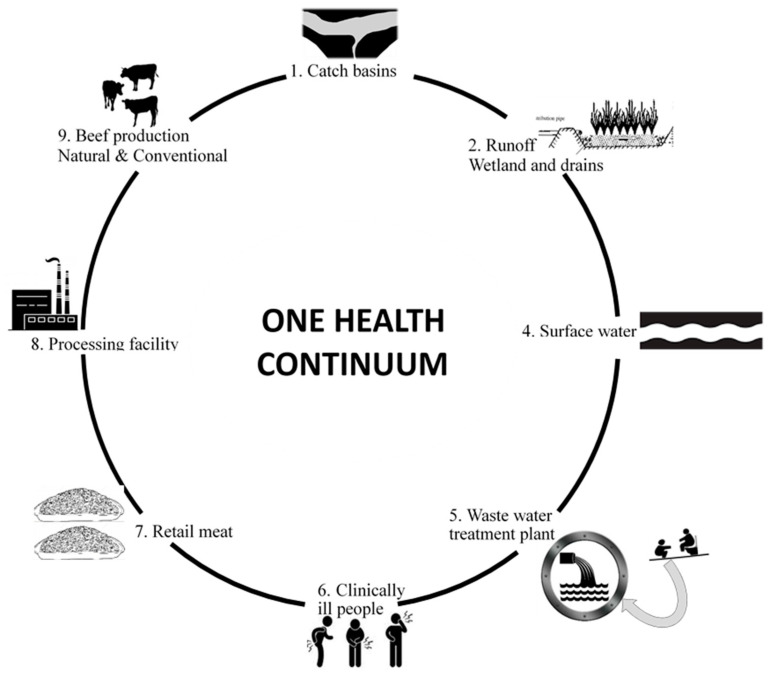
Details of sample type and source of *Escherichia coli* isolates investigated for antimicrobial resistance in a One Health study of the beef production system. Sample location/sites comprised composite fecal samples collected from penned cattle in feedlots A, B, C and D as well as water samples from catch basins. Environmental samples comprised constructed wetlands and a creek (adjacent to feedlot C). Wastewater influent and effluent were collected from two water treatment plants while samples at a beef processing facility were obtained from carcasses after hide removal, after final wash, from ground beef and retail meat. Human samples were obtained from blood, urine and abdomen samples collected from hospital patients in southern Alberta.

Sampling points and details of study area (Figure 1), associated sample collection and AMU records on farms are published elsewhere [33,34]. Samples were collected and transported to the lab on ice, with *E. coli* isolated within 24 h after arrival at the laboratory.

### 2.1. Study Area and Description of Sampled Matrices

#### 2.1.1. Fecal and Water Sampling from Cattle Feces, Catch Basins and Surrounding Streams

Four feedlots in Southern Alberta designated as A, B, and C used conventional production practices including the use of antimicrobials, while lot D was a commercial feedlot that employed both conventional and “raised without antimicrobials” practices. Feedlots had a one-time capacity of 15,000–40,000 cattle and were characterized by production conditions typical for western Canadian feedlots, with open air pens with dirt floors arranged side-by-side with central feed alleys. Animals used in this experiment were cared for in accordance with the Canadian Council of Animal Care [35]. All procedures and protocols used in this study were reviewed and approved by the University of Calgary’s Animal Care Committee (Protocol number AC14-0029). Fresh pen-floor fecal samples were collected every two months from April 2014 to April 2016. Twenty pens within each feedlot containing between 100 to 300 cattle were randomly selected, and the same pens were sampled throughout the two-year study. For each pen, a combined sample of 10 g of feces was obtained from 20 fecal pats to generate composite pen samples that were transported to the lab in Cary-Blair enteric transport medium (BD Canada, Inc., Mississauga, ON, Canada).

In each feedlot, runoff from the sampled pens drained into an adjacent catch basin. In feedlot C, water samples were also obtained from accumulated runoff periodically transferred into a constructed wetland and from an adjacent ephemeral creek into which the wetland drained. From mid-depth (≈0.75 m), 1 L of water was collected using a polyethylene bottle attached to a telescopic pole at four different locations per site, which were combined to generate a composite sample. For the wetland, one sample was generated by collecting and combining samples from four consistent locations throughout the study. Water samples were collected monthly from catch basins, the wetland and creeks from April to October of 2014 and 2015. Subsequently, samples from the creeks were collectively referred to as surface streams, while the wetland samples were categorized together with samples from the catch basin. Samples were not collected from these sources from November to March as they were often frozen. 

#### 2.1.2. Wastewater Treatment Plants (WWTP)

In tandem to farm sample collection, a composite of influent (post-grit tank) and effluent (just prior to release) sewage samples (1 L) was collected bimonthly from WWTP in Calgary and Medicine Hat from April 2014 to April 2016.

#### 2.1.3. Processing Plants

Protocols for beef processing plant and retail meat sample collection followed procedures published elsewhere [36,37]. Samples were obtained from ~100 cm^2^ areas of hides, beef trim and conveyers; 1000 cm^2^ areas of washed carcasses; and from the whole of the distal surface of each chilled side (~12 × 500 cm^2^). Carcasses or conveyer samples were collected by randomly swabbing the surface with a 2 × 2 sterile gauze pad (Millerdale Pharmacy, Dukal Corporation, Red Deer, AB, Canada) moistened with 0·1% *w/v* peptone water (Becton Dickinson Co., Sparks, MD, USA). For ground beef, 200 g was obtained by aseptically removing ground beef from 4–5 kg packs of coarsely ground chub. Samples were placed in sterile stomacher bags on ice and transported in a cooler to the lab. Processing plant samples and feedlot fecal samples did not arise from the same animals, although the feedlots sampled did send cattle to the same processing plant.

#### 2.1.4. Humans

Human clinical samples were randomly selected from blood, urine and abdominal samples from anonymous individual patients and did not require patient approval or a clinical use permit. The samples were collected during the same time period as those described above by the Division of Medical Microbiology, Calgary Laboratory Services (CLS) biorepository. This laboratory services about 1.5 million people in Calgary and the surrounding rural area. Clinical strains were isolated and confirmed from samples as described by Pitout et al [38,39].

### 2.2. Isolation of E. coli

Processing of samples from cattle feces, catch basin, surrounding streams and sewage treatment followed procedures detailed by Adator et al. [40] and Tymensen et al. [41] to obtain generic *E. coli* and ESC^r^. To isolate ESC^r^ from feedlot samples, 0.5 g of cattle feces was added to 4.5 mL *E. coli* broth-cefotaxime (2 μg/mL) and enriched overnight, followed by sub-culture onto MacConkey plates supplemented with 1 μg/mL of ceftriaxone (MilliporeSigma) [40]. Three distinct red/magenta lactose-fermenting colonies were subcultured onto tryptic soy agar (TSA)-ampicillin (32 μg/mL) (MilliporeSigma) and later archived at −80 °C. For catch basins and surrounding streams, ESC^r^ were isolated by membrane filtration using the US EPA Method 1603 [34,40]. To obtain generic *E. coli*, the same procedure, excluding enrichment and selective plates, was used. For samples from the beef processing plant (BProcessing), swabs were mixed with 10 mL of buffered peptone water (BPW), stomached for 2 min and incubated overnight at 37 °C. For ground beef, ~25 g of ground beef was incubated overnight at 37 °C in 225 mL of BPW. One mL from each resulting culture was added to 9 mL of *E. coli* enrichment broth containing a Durham tube and 2 µg/mL of cefotaxime, followed by overnight incubation at 37 °C and plating onto MacConkey agar containing 1 µg/mL of ceftriaxone. The plates were then incubated overnight at 37 °C. Samples from catch basin (CBasins), surface streams (SStreams), sewage treatment (MSewage) and humans (CHumans) were not enriched overnight prior to plating onto ceftriaxone-supplemented MacConkey plates.

Subsequently, pure putative *E. coli* colonies obtained from feedlots A, B and D, MSewage and BProcessing were subjected to indole test (Indole Spot Reagent, Hardy Diagnostics; Santa Maria, CA, USA). Three colonies positive for the indole reaction were deposited in Brain Heart Infusion (BHI) broth containing 15% glycerol and in Tris-EDTA (TE) buffer (pH 7.6) for DNA template extraction and stored at −80 °C. *E. coli* ATCC 25,922 and *M. haemolytica* 33,396 were included as indole positive and negative controls, respectively. *E. coli* was confirmed using the *arpA* gene for PCR before archiving [34]. A single colony obtained from antibiotic-supplemented plates with or without enrichment were collectively referred to as extended-spectrum cephalosporin-resistant *E. coli* (ESC^r^). ESC^r^ isolates originated from CFeces (*n* = 382), CBasins (*n* = 137), SStreams (*n* = 59), BProcessing (*n* = 4), MSewage (*n* = 98) and CHumans (*n* = 25), while generic *E. coli* isolates obtained on MacConkey agar originated from CFeces (*n* = 142), CBasins (*n* = 185), MSewage (*n* = 96), SStreams (*n* = 81) and BProcessing (*n* = 159).

### 2.3. Antibiograms

A comprehensive panel of antimicrobials was selected on the basis of: (i) use in beef production systems; (ii) category of medical importance to humans; (iii) adequate representation of antimicrobials in diverse classes; and (iv) use in phenotypic confirmation of extended-spectrum β-lactam -resistant *E. coli*. Resistance of ESC^r^ and generic *E. coli* to an antibiotic panel (ampicillin, amoxicillin/clavulanic, ceftiofur, ceftazidime, ceftazidime/clavulanic acid, streptomycin, neomycin, oxytetracycline, florfenicol, trimethoprim/sulfamethoxazole and sulfisoxazole) was examined using the Kirby–Bauer disk diffusion susceptibility method according to documents M100-S26 and VET-01S of the Clinical and Laboratory Standards Institute (CLSI) [42,43]. Briefly, *E. coli* isolates were retrieved from −80 °C and streaked onto TSA supplemented with 5% sheep blood (PB75, Dalynn Biologicals, Calgary, AB) and incubated overnight at 37 °C. A single colony from each plate was subcultured onto PB75, incubated overnight at 37 °C and then used for ASTs. Isolates were resuspended in saline and density was calibrated to a 0.5 McFarland turbidity standard. The suspension was streaked onto Mueller Hinton Kirby agar plates (PM90K) (Dalynn Biologicals). BD BBL™ Sensi-Disc™ (BD) (Becton, Dickinson and Company, Franklin Lakes, NJ, USA) or Thermo Scientific Oxoid (Dardilly, France) antimicrobial susceptibility disks were then placed onto the plates. The plates were incubated for 18 h and zone diameters of inhibition were recorded using BIOMIC V3 Microbiology System (Santa Barbara, CA) using standards set by the CLSI [42,43]. *E. coli* American Type Culture Collection (ATCC) 25,922, ATCC 35,218 and *Staphylococcus aureus* ATCC 29,213 were used as quality controls [42,44]. Putative extended-spectrum β-lactamase-resistant phenotypes were assigned if isolates showed an increased zone of inhibition of ≥5 mm for ceftazidime in combination with clavulanic acid versus the zone of inhibition obtained when tested against ceftazidime alone [42,45].

### 2.4. Genotypic Characterization of ESBL Resistance

A simplex and two multiplex PCR assays were used to screen 705 ESC^r^ using the primer sets and conditions previously validated elsewhere, with minor modifications. Multiplex 1 comprised *bla*_TEM_ [46], *bla*_SHV_ [46], *bla*_OXA_ [47], and *bla*_CMY_ [48], multiplex 2 comprised *bla*_CTX-M 1,2,9_ [47], while the simplex identified *bla*_CTX-M_ [46]. DNA template was prepared by heat lysing each *E. coli* colony in TE (pH 7.4) at 99 °C for 10 min and the lysate was centrifuged 21,130× *g* for 5 min (5424 R Eppendorf Centrifuge). Each 25 µL multiplex PCR reaction comprised 12.5 μL of 1× Qiagen HotStar Plus Multiplex PCR Master Mix (Qiagen GmbH, Hilden, Germany), 2.5 μL CoralLoad Concentrate, primer-specific concentration and 2 μL DNA template. Simplex reactions were comprised of 12.5 μL of Qiagen PCR Master Mix, 2.5 μL coral-load solution, primer-specific concentration and 2 μL DNA template. Amplification conditions for multiplex 1 were as follows: 95 °C for 15 min; 30 cycles of 94 °C for 1 min, annealing at 53 °C for 1 min, 72 °C for 1 min with a final extension for 10 min at 72 °C. For multiplex 2 and simplex reactions, annealing temperature was 60 °C. PCR products were subjected to electrophoresis in 1.5% agarose gel in 1X TAE buffer. *E. coli* strains previously sequenced with known β-lactamase genes of interest were used as positive controls and water was used as a no template control.

### 2.5. Data Management and Analysis

Descriptive analysis comparing the distributions of resistance (R), intermediate (I) and susceptible (S) *E. coli*, MDR and ESBL-resistant phenotypes as well as β-lactamase genotypes was performed with PROC FREQ (SAS software, version 9.4 SAS Institute, Cary, North Carolina, USA). Isolates were categorized as MDR if they showed resistance to antimicrobial agents in ≥2 different antimicrobial classes [49,50]. To compare the effect of isolate source on AMR in both ESC^r^ and generic *E. coli* sub-populations, univariate multinomial logistic regression models were fitted to the data using the SAS GLIMMIX procedure [13,51]. *E. coli* population (ESC^r^ or generic *E. coli*) was modeled as a random block factor, while antibiotic isolate source and their interaction were considered fixed effects. Differences in AMR for each antibiotic per source were expressed as odds ratios (OR); source-specific ORs were specified with a 95% confidence interval and declared significant at *p* < 0.05. Antimicrobials which were not modeled due to low prevalence of resistance or inability for the model to converge included neomycin in ESC^r^ and ceftiofur, ceftazidime, trimethoprim-sulfamethoxazole and neomycin in generic *E. coli*. Since not all *bla* genes are indicative of ESBLs, isolates were designated as cephalosporin-resistant, β-lactamase genotype-positive if they possessed one or more of *bla*_SHV_, *bla*_TEM_, *bla*_OXA_ and *bla*_CTXM_ and designated as a pAmpC genotype-positive if they possessed *bla*_CMY_. To examine the associations between the number or type of genes present and MDR phenotype per source, separate binary logit analyses were performed using logistic regression [52] and effects were considered significant at *p* < 0.05 [53]. For source effect analyses of AMR phenotypes and β-lactamase/pAmpC genes in ESC^r^, CHumans and MSewage ESC^r^ isolates were categorized together as human-associated *E. coli* designated HM (total *n* = 123). ESC^r^ BProcessing isolates were not included in the analyses due to the low number of isolates obtained.

## 3. Results

### 3.1. Antimicrobial Resistance in Extended-Spectrum Cephalosporin-Resistant E. coli and Generic E. coli 

**Figure 2:**
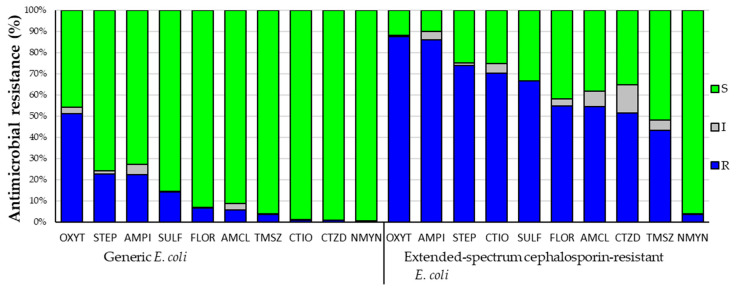
Antimicrobial resistance distribution among generic (*n* = 663) and extended-spectrum cephalosporin-resistant (*n* = 705) *Escherichia coli*. Legends: S—Susceptible; I—Intermediate; R—Resistance. OXYT—Tetracycline; STEP—Streptomycin; AMPI—Ampicillin; SULF—Sulfisoxazole; FLOR—Florfenicol; AMCL—Amoxicillin/clavulanic acid; TMSZ—trimethoprim/sulfamethoxazole; CTIO—Ceftiofur; CTZD—Ceftazidime; NMYN—neomycin.

A total of 705 ESC^r^ strains and 663 generic *E. coli* were examined for antimicrobial susceptibility to nine antimicrobial agents and two combinations. For ESC^r^, prevalence of resistance ranked as oxytetracycline (87.7%), followed by ampicillin (86%), streptomycin (73.9%), ceftiofur (70.2%), sulfisoxazole (66.8%), trimethoprim-sulfamethoxazole (43.4%) and neomycin (3.7%) (Figure 2). Generic *E. coli* exhibited the highest prevalence of resistance to oxytetracycline (51.1%), followed by streptomycin (22.6%), ampicillin (22.5%), sulfisoxazole (14.3%), trimethoprim-sulfamethoxazole (3.6%), ceftiofur (0.9%), ceftazidime (0.8%) and neomycin (0.5%) (Figure 2). In both populations, resistance to oxytetracycline and ampicillin was prevalent, while neomycin resistance was the least common. The overall prevalence of resistance to antimicrobials was higher (*p* < 0.001) for ESC^r^ than for generic *E. coli*.

Overall AMR prevalence among ESC^r^ from various sources (CFeces, CBasins, MSewage, BProcessing, MSewage, and CHumans) differed (*p* < 0.001; Table 1). ESC^r^ from CFeces exhibited high levels of resistance to oxytetracycline (98.4%) followed by ampicillin (96.3%), streptomycin (89.0%), sulfisoxazole (85.1%), ceftiofur (78.2%), florfenicol (77.2%), amoxicillin clavulanate (71.7%), ceftazidime (65.4%), trimethoprim-sulfamethoxazole (51.0%) and neomycin (4.9%; Table 1). Although less common than in CFeces, resistance to oxytetracycline, ampicillin and streptomycin was also most prominent in CBasins and SStreams isolates. Resistance to neomycin was rare, regardless of isolate source. ESC^r^ from MSewage and CHumans did not exhibit specific similarities in AMR trends. In MSewage, ESC^r^ exhibited the highest resistance to ampicillin (99%) and ceftiofur (93.9%), while clinical isolates from humans showed the highest resistance to ampicillin (100%), ceftiofur (92%) and oxytetracycline (84%; Table 1).

Comparison of source among ESC^r^ (Table 1; Table S1a–i; Figure S1) revealed that for all antimicrobials, resistance was higher in CFeces than in CBasins and SStreams isolates (*p* ≤ 0.002), which did not differ (*p* > 0.2). CFeces isolates were more (*p* ≤ 0.001) resistant to oxytetracycline (98.4%), streptomycin (89.0%), sulfisoxazole (85.1%), florfenicol (77.2%), amoxicillin/clavulanate (71.7%) and ceftazidime (65.4%) than HM isolates (CHumans and MSewage isolates combined), which averaged 70.1%, 46.5%, 53.9%, 6.6%, 30.9% and 31.9%, respectively. In contrast, ceftiofur resistance was higher (*p* = 0.01) in HM than CFeces isolates. HM isolates also showed higher (*p* ≤ 0.01) resistance than CBasins and SStreams to ampicillin, ceftiofur, ceftazidime and trimethoprim-sulfamethoxazole. Florfenicol resistance levels were higher in CBasins (45.3%) and SStreams (28.8%) isolates than in HM isolates (6.6%). For almost all antimicrobials, HM isolates differed in AMR prevalence from those obtained directly from cattle or surrounding environments (Table 1; Table S1a–i). Additionally, an antibiotic*source interaction (*p* < 0.001) was observed for the prevalence of resistance to some antimicrobials, associated specifically with isolates from CBasins, CFeces and HM. Synergistic interactions associated with slightly higher resistance to specific antimicrobials included HM*amoxicillin/clavulanate, sulfisoxazole (*p* ≤ 0.001), both CBasins and HM*ampicillin, ceftazidime and ceftiofur (*p* ≤ 0.001) and CFeces*florfenicol (*p* < 0.04), while isolates from all three of these sources were associated with higher resistance to trimethoprim-sulfamethoxazole (*p* ≤ 0.001).

Generic *E. coli* isolates from all environments (CFeces, MSewage, SStreams, CBasins and BProcessing) were the least resistant to neomycin (0.0% to 1.2%), followed by ceftazidime (0.0% to 2.1%). Resistance to individual antimicrobials was higher for generic isolates from CFeces and CBasins (Table 1; Figure S1). Similar resistance trends in CFeces (oxytetracycline, 88.7% > streptomycin, 41.6% > sulfisoxazole, 25.4%), but at lower frequencies were observed in CBasins (oxytetracycline, 72.4% > streptomycin, 29.2% > sulfisoxazole, 18.4%), with low resistance to ceftazidime, ceftiofur and neomycin (Table 1; Figure S1). In BProcessing, MSewage and SStreams, resistance to ampicillin (44.0%, 21.9% and 8.6%) and oxytetracycline (19.5%, 12.5% and 44.4%) was most prevalent (Table 1). Independently, the source of generic *E. coli* did not affect the prevalence of AMR (*p* = 0.998), but an antibiotic*source interaction was observed (*p* < 0.001). Oxytetracycline resistance in CFeces (88.7%) was higher than BProcessing (19.5%; *p* = 0.03) and MSewage (12.5%; <0.001) (Table 1; Figure S1).

For generic isolates, ampicillin resistance was higher in BProcessing (44.0%; *p* < 0.001) and MSewage (21.9%; *p* = 0.01) than in CBasins isolates. Resistance to amoxicillin/clavulanate in CBasins isolates was also slightly higher than in CFeces and SStreams (*p* ≤ 0.02), but less than BProcessing isolates (*p* = 0.004). Streptomycin resistance was higher in CFeces (41.5%; *p* ≤ 0.0013) than MSewage and CBasins isolates, while sulfisoxazole resistance was higher (*p* = 0.04) for CFeces than CBasins isolates. Prevalence of florfenicol resistance did not differ across most sources (*p* ≥ 0.09), with no resistance to this antibiotic detected in isolates from MSewage. For most antimicrobials, prevalence of resistance in generic *E. coli* population did not differ between CBasins, SStreams and MSewage (Table 1; Table S1j–o; Figure S1).

### 3.2. Distribution of Multidrug Resistance in Extended-Spectrum Cephalosporin-Resistant E. coli and Generic E. coli

Altogether, 88.8% of ESC^r^ were MDR, with the majority resistant to six different classes of antimicrobials (Table 2). Generic *E. coli* isolated on antibiotic-free media exhibited MDR in 26.7% of the isolates (Table 2). Altogether, ESC^r^ collected from MSewage (96.9%), CHumans (96%), CFeces (97.1%) and BProcessing (100%) exhibited high MDR, with the least MDR observed in isolates from SStreams (54.2%) (Table 2; Table S3 and Figure S2). With generic *E. coli*, prevalence of MDR was highest in CFeces (45.1%), CBasins (34.6%), SStreams (23.5%), MSewage (13.6%) and BProcessing (10.7%) (Table 2; Figure S2). Confirmed ESBL phenotypes were more prevalent in ESC^r^ isolates from CHumans (64%), followed by MSewage (48%), CFeces (22.5%), SStreams (15.3%) and CBasins (10.9%), with no ESBL phenotypes identified within BProcessing (Table 3). The most common MDR profiles in ESC^r^ were oxytetracycline, streptomycin and sulfisoxazole with the majority of this group exhibiting the phenotypes AMPI–CTZD–AMCL–CTIO–STEP–SULF–FLOR–OXYT–TMSZ (41.3%) and AMPI–CMCL–CTZD–CTIO–STEP–SULF–FLOR–OXYT (35.2%) (Table S2). For generic *E. coli* the most common MDR profiles were to OXYT, AMPI and STEP. Combined, 24.7% of the ESC^r^ were positive for ESBL phenotype, whereas only 0.6% (*n* = 3 MSewage; *n* = 1 from BP) of the generic *E. coli* isolates exhibited true ESBL phenotypes (data not shown). Hence, confirmed phenotypic ESBL *E. coli* were not found among generic *E. coli* isolated from feedlot environments.

### 3.3. AMR Determinants of Extended-Spectrum Cephalosporin-Resistant E. coli

Overall, 40.9% of the ESC^r^ population possessed at least one cephalosporin-resistant β-lactamase gene, with 81.3% of isolates possessing pAmpC *bla*_CMY_. All (100%) *E. coli* isolates of human origin possessed ≥1 cephalosporin-resistant β-lactamase gene, followed by isolates from MSewage (80.6%), CFeces (35.3%), CBasins (27.0), SStreams (22.0%) and BProcessing (0%) (Table S3). Trends of ESBL phenotypes followed a similar pattern as we found for β-lactamase genotypes CHumans > MSewage > CFeces > CBasins > SStreams > BProcessing, but prevalence of ESBL phenotypes were generally lower than the identification of β-lactamase determinants (Table 3; Table S3), with a higher diversity of genes occurring in MSewage isolates (Table 3; Table S3). However, no apparent trends were observed between β-lactamase and pAmpC genotypes and MDR phenotypes relative to source, although the top three sources (CHumans, MSewage and CBasins) remained consistent for both MDR and β-lactamase genotypes (Table 3, Figure S2 and Table S3). Assessment of individual *bla* genes per source revealed that most were *bla_CTX_*_-*M*_ genes. Extended-spectrum cephalosporin-resistant *E. coli* from CFeces harbored *bla_CTX-M_* (25.4%), followed by *bla*_CTX-M-1_ (18.6%), *bla_TEM_* (15.4%), *bla_CTX-M-9_* (5.8%), *bla*_CTX-M-2_ (5.2%) and *bla_SHV_* (1.8%), while *bla_OXA_* was not detected. Clinical isolates tended to be higher in *bla_CTX-M_* (96%) and *bla*_CTX-M-1_ (72%), followed by *bla_TEM_* (48%) and *bla_OXA_* (28%) (Table 3). For the remaining sources, MSewage isolates also harbored *bla_CTX-M_* (67.3%), SStreams isolates *bla_CTX-M_* and *bla_TEM_* (15.3% each) and CBasins isolates *bla_TEM_* (19.7%). SStreams and CBasins isolates also exhibited the highest prevalence of *bla_CMY_* at 93.2% and 91.2% respectively, with a lower level of this determinant (32%) in CHumans isolates.

Generally, the source of isolate was found to influence MDR phenotypes in the ESC^r^ population (*p* < 0.004), although the exact or relative number of genes did not influence MDR. Additionally, when the effect of the presence or absence of a single gene was examined, statistical analyses revealed that MDR prevalence was not affected by the presence of just a single gene. Examination of the effect of specific *bla*/pAmpC gene types also revealed that the presence of the genes (SHV, TEM, OXA, CTX-M, CTXM 1, CTX-M 2, CTX-M 9 and CMY) did not influence the prevalence of MDR.

## 4. Discussion

### 4.1. Overall and Longitudinal Antimicrobial Resistance

In this investigation, enrichment procedures were used to increase the likelihood of isolating extended-spectrum cephalosporin-resistant *E. coli* (ESC^r^) within the beef production system. This study is the first to comprehensively assess AMR ESC^r^ and generic *E. coli* in beef production systems from the perspective of a One Health continuum within the same geographical region.

In the present study, oxytetracycline, ampicillin and streptomycin resistance were the highest in both ESC^r^ and generic *E. coli*. The generally high levels of AMR in ESC^r^ as opposed to generic *E. coli* is a potential reflection of the enrichment procedure selecting for ESC^r^. Genes such as *bla_SHV_*, *bla_TEM_* and *bla*_CTX-M_ are often borne on mobile genetic elements that code for resistance to multiple antimicrobials [54,55,56]. Although enrichment for ESC^r^ was expected to only isolate β-lactamase resistant *E. coli*, 4.1% of isolates were susceptible to ampicillin. Factors such as silencing of resistance genes in *E. coli* [57] and the loss of plasmids in the absence of selective pressure may explain this finding [58,59,60].

Overall, high prevalence of AMR in ESC^r^ agrees with reports of resistance to 3rd generation cephalosporin *E. coli* (3GC^r^) isolated from the beef processing continuum [15], as well as wild birds and associated nearby water sources [61]. Schmidt et al. [15] reported AMR for ampicillin, ceftiofur, tetracycline and streptomycin (100%, 100%, 97.3% and 65.3%) in 3GC^r^ *E. coli,* while our study found that 85.6%, 69.7%, 87.1%, 73.4% and 66.4% of ESC^r^ were resistant to ampicillin, ceftiofur, oxytetracycline, streptomycin and sulfisoxazole, respectively. Similarly, in generic *E. coli* isolated from conventionally raised beef cattle [62] and from dairy cattle, swine, horses, sheep, goats, chickens, cats, dogs, deer, ducks, geese, human sewage and surface water, resistance to tetracycline, streptomycin and sulfisoxazole was most common [13]. 

Significant differences in AMR profiles associated with various isolate sources coincide with findings by Ojer-Usoz et al. [63] where they investigated ESBL *E. coli* from food, wastewater, rivers, farms and humans and found higher cefepime (a 4th GC) resistance in food and human isolates (94.2% and 94.6%, respectively), compared to farm isolates (2.0%). In our study, prevalence of AMR was higher in CFeces compared to CBasins, SStreams, BProcessing and HM (MSewage and CHumans combined), with the exception of ampicillin, ceftiofur and trimethoprim/sulfonamide. In North America and the United Kingdom, tetracyclines, penicillins and aminoglycosides tend to be among the major classes of antimicrobials used in beef production [7,64]. Widespread use of tetracyclines in livestock increases tetracycline-resistant *E. coli* in feces [62,65]. In this study, the conventional feedlots used tetracycline so it is not surprising that tetracycline resistance was the highest in *E. coli* isolated at the point source of utilization and declined with movement from CFeces to CBasins and surrounding water ways.

Runoff water from pens collects in CBasins, and from there it may enter the surrounding environments, resulting in the prevalence of resistance to oxytetracycline, ampicillin, and streptomycin and neomycin being similar in *E. coli* isolated from these environments. Considering that AMR prevalence of CFeces > CBasins > SStreams isolates, CFeces may serve as a point source from which antibiotic residues as well as AMR bacteria and genes may be disseminated. The decreasing AMR proportions in CBasins and SStreams could reflect decreasing selective pressure as a result of lower antibiotic residue concentrations in these environments as compared to CFeces [66,67]. According to Sayah et al. [13], similarities of AMR patterns in different farm sources suggest a common source of resistant bacteria, an assertion that agrees with our findings. Likewise, Ibekwe et al. [68] found that elevated levels of AMR *E. coli* in surface water coincided with nonpoint sources of fecal contamination from both agricultural and human sources.

ESC^r^ from MSewage and CHumans differed in the top-three resistant phenotypes (ampicillin > ceftiofur > oxytetracycline) as compared to ESC^r^ (oxytetracycline > ampicillin > streptomycin) isolated from cattle. Symptomatic correlations between AMR and selective antibiotic pressures are expected when both antimicrobials and bacterial isolates are derived from a common source, as is the case when human feces enter surface waters in the form of sewage effluent [69]. The similarity in AMR phenotypes in MSewage and CHumans *E. coli* isolates is consistent with this observation. 

Generic *E. coli* isolated from CFeces, CBasins, SStreams and MSewage were most often resistant to oxytetracycline, streptomycin, sulfisoxazole and ampicillin. Antibiograms of CFeces isolates, which aligned with those from CBasins suggests that catch basins restricted the flow of AMR *E. coli* into surrounding surface waters. Generic BProcessing isolates were resistant to ampicillin, oxytetracycline and amoxicillin-clavulanic acid, likely reflecting the frequent use of penicillins and tetracyclines in beef production [13]. 

### 4.2. Resistance to Antimicrobial Agents of Clinical Relevance Relative to Source

#### 4.2.1. Oxytetracycline

High prevalence of resistance to oxytetracycline in *E. coli* has been reported in several other studies related to beef cattle and the environment [13,62,66,68]. This finding is not surprising as tetracycline is a first-line antibiotic used in beef cattle to control liver abscesses and bovine respiratory disease [7,13]. Interestingly, oxytetracycline resistance in generic MSewage isolates in this study was less than in a study by Ibekwe et al. [68], where the highest tetracycline resistance was found in isolates from WWTPs (~27%), followed by urban and agricultural runoff. 

#### 4.2.2. Penicillins

Schmidt et al. (2013) and Volkova et al. (2012) reported 100% ampicillin and 64.7% amoxicillin resistance in 3GC^r^ *E. coli* from CFeces, hides, pre-evisceration and final carcasses. In our study, 96.3% and 71.7% of ESC^r^ isolates from CFeces were resistant to ampicillin and amoxicillin/clavulanic acid, respectively, while all human isolates in our study were resistant to ampicillin. A USDA report examining *E. coli* isolates from 1950–2002 consistently found more resistance to antimicrobials such as penicillin, which have been used for decades in clinical and veterinary medicine [70]. In another report, ≤2% of 600 isolates from agricultural sources and urban runoff were resistant to amoxicillin-clavulanic [68]. Consequently, it can be speculated that there are other prevailing conditions such as antibiotic residues at the point of isolation which may impact ampicillin and amoxicillin/clavulanic acid resistance in specific populations.

Resistance to ampicillin and amoxicillin/clavulanic acid was consistently higher in both ESC^r^ and generic BProcessing isolates. As such, it makes sense to surmise that irrespective of sub-population, β-lactamase-resistant *E. coli* could potentially contaminate beef products. 

#### 4.2.3. Aminoglycoside

Streptomycin resistance was found to range between 44% (CHumans) and 89.1% (CFeces) in ESC^r^. Coincidentally, high streptomycin (84.2%) and low kanamycin resistance (5.3%) were reported in generic *E. coli* from surface water. [66]. It is interesting to note that our isolates showed neomycin resistance ranging between 0% and 5.1% for ESC^r^, while very few generic *E. coli* were neomycin resistant. In generic isolates, streptomycin resistance ranged from 5.7% in BProcessing isolates to 41.5% in CFeces. These results are consistent with the observation of very low neomycin and higher streptomycin resistance in *E. coli* from feedlot cattle [62]. In the current investigation, CHumans ESC^r^ exhibited low prevalence of streptomycin and neomycin resistance, a finding consistent with those of others [71]. *E. coli* from CFeces and CBasins in both sub-populations showed high resistance to streptomycin even though it was not administered to cattle, suggesting possible co-selection for this resistance when other antimicrobials such as tetracyclines are administered [70,72,73].

#### 4.2.4. Trimethoprim/Sulfonamide

Trimethoprim-sulfamethoxazole is a first-line therapy for acute urinary tract infection (UTI), one of the most common infections in outpatients [74,75]. We found sulfamethoxazole-trimethoprim resistance to be rare in both generic and ESC^r^, as have others [13]. Sulfamethoxazole-trimethoprim resistances between 0.0% and 2.2% and sulfisoxazole between 0.0% and 13.98% have been reported in generic *E. coli* from livestock, companion animals, farm environments, surface water and human septage [13], compared to 1.9% (BProcessing) to 6.3% (CFeces) in generic *E. coli* in our study. Since this antibiotic is mainly used in human medicine, it is unsurprising that clinical human isolates demonstrated a higher prevalence of resistance than those from the beef production system. 

#### 4.2.5. Phenicol

It has been reported that florfenicol use in livestock co-selects for chloramphenicol resistance [76,77,78]. Florfenicol is approved for use in treating BRD. Donaldson et al. [76] reported 93% florfenicol and 94% chloramphenicol resistance in 3GC^r^ *E. coli* isolated from dairy cattle, while 92% of *E. coli* isolated at necropsy or from fecal samples of calves with diarrhea were florfenicol resistant [79]. In the present study, florfenicol resistance in ESC^r^ was 77.4% in CFeces, while it was low ≤ 45% in isolates from other environments. Similarly, *Escherichia/Shigella*, *Pseudomonas*, *Sphingobactaria*, and *Brevundimonas* obtained from a university hospital outlet and municipal sewage inlet on sulfamethoxazole/trimethoprim and streptomycin-selective plates exhibited florfenicol resistance in ≤45% of the isolates [80]. Results from our study suggest that florfenicol resistance in *E. coli* was generally infrequent in human-associated sources, likely due to limited use of phenicols in humans.

#### 4.2.6. Third-Generation Cephalosporins and ESBL Phenotype Distribution

In cattle feedlots, commensal 3GC^r^ *E. coli* represent a sub-population of generic *E. coli* associated with hide and fecal populations [81,82]. We observed that ceftiofur resistance (93.9%) in MSewage ESC^r^ isolates was substantially higher than in all other sources except for CHumans isolates (92%). Ceftiofur (38.9%) and ceftazidime (27.1%) resistance in SStreams ESC^r^ isolates was comparably lower than cefotaxime-resistant (77%) ESBL *E. coli* isolated from rivers and lakes in Switzerland [83]. Schmidt et al. [81] and Volkova et al. [82] also reported ceftiofur and ceftriaxone resistance of 100% in 3GC^r^ *E. coli* from cattle feces, hide and carcasses as compared to 66.7% of isolates from BProcessing and 65.5% from CFeces being resistant to ceftazidime in our study. It is possible that differences in the prevalence of 3GC resistance within ESC^r^ populations are attributable to differences in isolation methods, including the specific type and concentration of antimicrobial used for enrichment. The use of enrichment broths containing cephalosporins may also enhance bacterial conjugation and exchange of resistance plasmids between bacteria [28]. 

The β-lactam antimicrobials including cephalosporins are routinely used for treating bacterial infections in humans so that hospitals are a significant source of cephalosporin antimicrobials in wastewater [84]. Korzeniewska et al. [84] found that the majority of generic *E. coli* in three different hospital sewage plants were resistant to cefotaxime and ceftazidime, a result similar to ours with MSewage ESC^r^ isolates, although this contrasted the low resistance in our generic *E. coli* isolates. ESBL *E. coli* were readily obtained from MSewage even without enrichment with ceftriaxone. Discharge of untreated sewage containing antimicrobial residues and resistant bacteria from humans and animals may contribute to the emergence and spread of AMR bacteria in aquatic environments [80]. It is possible that antimicrobial residue levels in MSewage exert sufficient selective pressure for the development of MDR phenotypes. It was not surprising that clinical isolates obtained without enrichment showed the highest ESBL phenotype, as β-lactams are widely used in Canadian clinics.

Phenotypic ESBL isolates were not detected in BProcessing samples, an observation that agrees with others that did not detect ESBL *E. coli* in raw milk or minced beef, despite enrichment [85]. Furthermore, these observations suggest that there is a decrease in ESBL bacteria after defecation as one moves down the production chain [86]. The overall low levels of phenotypic ESBL *E. coli* reported suggests that without enrichment, negligible levels would have been detected within the beef production environment.

### 4.3. Prevalence of Multidrug Resistance

Of the 88.8% MDR ESC^r^ in the current study, most (45.1%) were resistant to antimicrobials in six different drug classes. The most common MDR patterns in ESC^r^ involved combinations of oxytetracycline, ampicillin, streptomycin, ceftiofur, florfenicol and sulfisoxazole, whereas oxytetracycline, streptomycin and sulfisoxazole were common in generic *E. coli*. Others have also found high levels of oxytetracycline, ampicillin and streptomycin resistance in generic *E. coli* isolates from feedlot cattle, farm environments, human septage and surface water [13,62]. In concordance with results from Ojer-Usoz et al. [63], MDR ESC^r^ exceeded 60% in all sources. Ojer-Usoz et al. [63] found levels of MDR in ESBL *E. coli* ranging between 58% and 86.2% in food, WWTP, rivers, farms and humans. Numerous investigators note that the administration and presence of even a single antimicrobial can select for MDR strains in both humans and animals [87,88,89]. Consequently, it is likely that the high MDRs reflect long-term exposure of bacteria to specific antimicrobials [87]. Long-term tetracycline use confers resistance to other antimicrobial agents via co-selection, as *tet* genes and other resistance genes often share common integrons, plasmids or transposons [65,90]. 

### 4.4. AMR Determinants Associated with Extended-Spectrum Cephalosporin-Resistant E. coli

#### 4.4.1. Occurrence of Bla Genes

Per source, *bla* genes from CFeces, MSewage and CHumans isolates were mostly *bla*_CTX-M_ in agreement with studies conducted in Canada [27,32] and other countries [91,92]. *Enterobacteriaceae* possessing *bla*_CTX-M_ variants have been isolated from food [93], poultry [94], companion animals [95,96], clinical settings [97,98,99] and wastewater [100,101]. Most clinical isolates in the current study possessed *bla*_CTX-M_ (96%) a finding that agrees with Ojer-Usoz et al [63]. In MSewage isolates, we also detected *bla*_CTX-M_ (67.3%), a result that aligns with clinical and human sewage isolates obtained by others [84,102]. 

The SHV- and TEM-type genes have also been detected in livestock and meat products [63,103], while OXA has been rarely reported in livestock. Brinas et al. [104] detected SHV- or OXA-type β-lactamases in only 3% of 124 ampicillin-resistant *E. coli* recovered from food and feces of healthy animals and humans. This is only slightly higher than the level of SHV (1.4%) we observed in ESC^r^. Interestingly, the OXA-type genes in our study were unique to MSewage and clinical isolates, while SHV-type were specific to CFeces and MSewage. Agga et al. [11] also reported a higher diversity of resistance genes in municipal wastewater than in livestock environments. In wastewater, the commonality of homologous regions within complex DNA elements enhances recombination [105,106], a response that may account for the high diversity of *bla* genes in MSewage. The complete absence of *bla_OXA_* in our isolates from the beef production environment agrees with Ojer-Usoz et al. [63], who found *bla*_OXA-1_ (20%) only in clinical *E. coli* isolates and not in isolates from food, farm or aquatic environments. OXA-type β-lactamases have often been reported in clinical *Enterobactreacea* around the world as well as from WWTP, with some variants implicated in hospital outbreaks [107,108,109].

#### 4.4.2. Occurrence of AmpC Gene

Few investigations have assessed the frequency of *bla_CMY_* genes in *E. coli* isolated from food and environmental matrices as compared to isolates obtained from clinical settings. Of ESC^r^ from cattle sources, we found *bla_CMY_* ranging from 87.2% to 93.2%, in contrast to the lower prevalence of 32% to 50% in human sources. In Canada, *bla_CMY_* has mostly been detected in clinical or hospital-associated isolates and infrequently in food-associated *Enterobacteriaceae*, while others have suggested that food sources may be an emerging concern [110,111,112].

## 5. Conclusions

Overall, AMR profiles of *E. coli* from the various sources reflected corresponding antimicrobial use in those segments of the continuum. A point source effect on AMR occurrence, where sources with antibiotic use reflected high AMR *E. coli* was further underscored by the similarity of AMR patterns between CFeces and CBasins. As such, continuing to challenge both the human medical and veterinary communities to monitor use protocols and improve antimicrobial stewardship practices in line with community, national and global AMR reduction goals. Continuous monitoring of critically important antimicrobials such as neomycin should be considered as a means of detecting early changes in resistance trends and onset of AMR emergence. *E. coli* isolation with enrichment enhanced the sensitivity of detecting ESBL-producing bacteria. ESBL phenotypes within ESC^r^ were more frequently associated with human than cattle sources. In generic *E. coli*, MDR was lowest in BProcessing isolates, suggesting that strategies employed during beef processing reduce the risk of MDR isolates in final meat products. 

## Figures and Tables

**Table 1:** Trends of antimicrobial resistance prevalence in extended-spectrum cephalosporin-resistant *E. coli* (ESC^r^) and generic *E. coli* populations with specificity to sample origin, expressed as percentages.

Prevalence (%) of antimicrobial resistance in extended-spectrum cephalosporin *E. coli* per source	**Environment**	**OXYT**	**AMPI**	**STEP**	**SULF**	**CTIO**	**FLOR**	**AMCL**	**CTZD**	**TMSZ**	**NMYN**
	CFeces	98.4	96.3	89	85.1	78.3	77.2	71.7	65.4	51	5
	CBasins	88.3	62.8	64.2	43.1	39.4	45.3	33.6	33.6	27	0.7
	SStreams	71.2	45.8	52.5	44.1	39	28.8	23.7	27.1	27.1	0
	BProcessing	75	100	75	75	100	75	100	75	0	0
	MSewage	56.1	99	49	43.9	93.9	9.2	41.8	43.9	46.9	5.1
	CHumans	84	100	44	64	92	4	20	20	52	4
Pair wise comparison of resistance prevalence for sources of extended-spectrum cephalosporin *E. coli*	CBasins vs. CFeces	CB < CF	CB < CF	CB < CF	CB < CF	CB < CF	CB < CF	CB < CF	CB < CF	CB < CF	nd
	CBasins vs. HM*	CB > HM	CB < HM			CB < HM	CB > HM		CB < HM	CB < HM	
	CBasins vs. SStreams										
	CFeces vs. HM*	CF > HM		CF > HM	CF > HM	CF < HM	CF > HM	CF > HM	CF > HM		
	CFeces vs. SStreams	CF > SS	CF > SS	CF > SS	CF > SS	CF > SS	CF > SS	CF > SS	CF > SS	CF > SS	
	HM* vs. SStreams		HM > SS		HM > SS	HM > SS	SS > HM	HM > SS	HM > SS	HM > SS	
Prevalence (%) of antimicrobial resistance in generic *E. coli* per source	CFeces	88.7	13.4	41.5	25.4	0.7	12	1.4	0.7	6.3	0
	CBasins	72.4	17.3	29.2	18.4	1.1	9.7	1.6	1.1	2.2	1.1
	SStreams	44.4	8.6	22.2	12.3	0	8.6	1.2	0	7.8	1.2
	BProcessing	19.5	44	5.7	3.8	0	1.3	18.9	0	1.9	0
	MSewage	12.5	21.9	10.4	9.4	3.1	0	1	2.1	4.2	0
Pair wise comparison of resistance prevalence for sources of generic *E. coli*	CBasin vs. CFeces	CB < CF				nd			nd	nd	nd
	CBasins vs. BProcessing	CB > BP	CB < BP	CB > BP	CB > BP			CB < BP			
	CBasins vs. MSewage	CB > MS		CB > MS							
	CBasins vs. SStreams	CB > SS									
	CFeces vs. BProcessing	CF > BP	CF < BP	CF > BP	CF > BP		CF > BP	CF < BP			
	CFeces vs. MSewage	CF > MS		CF > MS	CF > MS						
	CFeces vs. SStreams	CF > SS		SS < CF							
	BProcessing vs. MSewage		BP > MS								
	BProcessing vs. SStreams	BP > SS	BP > SS	BP < SS				BP > SS			
	MSewage vs. SStreams	MS < SS									

In the ESC^r^ population, *E. coli* resistance to individual antibiotics differ across sources (*p* < 0.001), whereas in the generic population, *E. coli* did not differ across sources (*p* < 0.99), although differences were observed per source*antibiotic interaction in both populations (*p* < 0.001). Pairwise comparisons display significant differences in antibiotic resistance between locations (0.0 ≤ *p* ≤ 0.03 for ESC^r^ *E. coli*, while 0.0 ≤ *p* ≤ 0.04 for generic *E. coli*; Table S3). CF—cattle feces, CB—catch basins, SS—Surface streams, BP—Beef processing, MS—Municipal sewage, CH—Human clinical isolates while HM* represents the total of human clinical and municipal sewage isolates. nd represents antibiotics which were not modeled due to low prevalence of resistance and as a consequence the model did not converge.

**Table 2:** Distribution of multidrug resistance in extended-spectrum cephalosporin-resistant *E. coli* and generic *E. coli* population per source.

*E. coli* Population	Multidrug Resistance (%)	R6	R5	R4	R3	R2	R1	S
Extended-spectrum cephalosporin-resistant *E. coli* (*n* = 705)	Overall MDR in ESC^r^ *E. coli*	45.2	17.9	2.3	15.6	7.8	7.4	3.8
	Cattle feces (*n* = 382)	64.4	19.6	0.3	12.0	0.8	2.6	0.3
	Catch basin (*n* = 137)	34.3	6.6	0.0	19.0	13.1	19.7	7.3
	Surface streams (*n* = 59)	28.8	6.8	0.0	11.9	6.8	18.6	27.1
	Municipal sewage (*n* = 98)	5.1	29.6	12.2	22.4	27.6	3.1	0.0
	Beef processing (*n* = 4)	75.0	0.0	0.0	0.0	25.0	0.0	0.0
	Human (*n* = 25)	4.0	36.0	12.0	36.0	8.0	4.0	0.0
Generic *E. coli* (*n* = 663)	Overall MDR in generic *E. coli*	0.6	2.9	4.4	9.2	9.7	37.9	35.4
	Cattle feces (*n* = 142)	0.7	4.9	7.7	14.1	17.6	45.1	9.9
	Catch basin (*n* = 185)	1.6	3.8	4.9	11.9	12.4	40.5	24.9
	Surface streams (*n* = 81)	0.0	3.7	4.9	7.4	7.4	21.0	55.6
	Municipal sewage (*n* = 96)	0.0	0.0	5.2	5.2	3.1	15.6	70.8
	Beef processing (*n* = 159)	0.0	1.3	0.0	5.0	4.4	50.3	39.0

S—% susceptible; R—% resistance to specific number of antimicrobial classes, represented 1–6. Total of 88.8% of extended-spectrum cephalosporin-resistant isolates showed MDR phenotypes and were resistant to antimicrobials belonging to at least two different antimicrobial classes, while 26.7% of generic *E. coli* isolates were MDR.

**Table 3:** Proportion of true ESBL phenotypes and β-lactamase among extended-spectrum cephalosporin-resistant *E. coli* isolates from multiple sources.

Source	Phenotypic Confirmatory Test		β-Lactamase Genes							
Sources	ESBL	non-ESBL	SHV	TEM	OXA	CTXM	CTXM 1	CTX-M 2	CTX-M 9	CMY
Human	64.0	36.0	0.0	48.0	28.0	96.0	72.0	24.0	24.0	32.0
Municipal sewage	48.0	52.0	3.1	34.7	14.3	67.3	33.7	19.4	28.6	50.0
Cattle feces	22.5	77.5	1.8	15.4	0.0	25.4	18.6	5.2	5.8	87.2
Catch basin	11.7	88.3	0.0	19.7	0.0	11.7	11.7	0.0	0.0	91.2
Surface water	15.3	84.7	0.0	15.3	0.0	15.3	15.3	0.0	0.0	93.2
Processing plant	0.0	100.0	0.0	0.0	0.0	0.0	0.0	0.0	0.0	50.0

Total ESBL prevalence was 24.7% in extended-spectrum cephalosporin-resistant *E. coli* sub-population (*n* = 705). ESBL phenotypic confirmation was achieved using the combination disk method such that a ≥5-mm increase in zone diameter for ceftazidime tested in combination with clavulanate vs the zone diameter of ceftazidime when tested alone equaled confirmed phenotypes.

## Supplementary Materials

The supplementary materials are available online at https://www.mdpi.com/2076-2607/8/6/885/s1.

## Abbreviations

CFeces	Cattle feces
CBasins	Catch basins
SStreams	Surrounding streams
BProcessing	Beef processing plants
MSewage	Municipal sewage treatment
CHumans	Humans clinical isolates
CLSI	Clinical and Laboratory Standards Institute
AMR	Antimicrobial resistance
MDR	Multidrug resistance
ESBL	Extended-spectrum beta-lactamase
ESC^r^	Extended-spectrum cephalosporin-resistant *E. coli*
3GC^r^	Third generation cephalosporin resistance
HM	Human and Municipal Sewage isolates combined
WWTP	Wastewater treatment plant
