# Supplementary material for: A One Health Comparative Assessment of Antimicrobial Resistance in Generic and Extended-Spectrum Cephalosporin-Resistant Escherichia coli from Beef Production, Sewage and Clinical Settings
Source: Microorganisms. 2020 Jun 11;8(6):885. doi: 10.3390/microorganisms8060885 (PMC7355928; doi:10.3390/microorganisms8060885)
Supplement: Supplementary file 1 [file microorganisms-08-00885-s001.pdf]

SUPPLEMENTARY TABLES AND FIGURES

Table S1a. Evaluation of differences in oxytetracycline resistance occurring among extended-spectrum cephalosporin resistant *E. coli* originating from multiple segments of the One Health continuum

Sample	Variable	Odds ratio	95% CI	P-value
CB	CF	0.95	5.37 - 73.91	<.0001
CB	HM	0.19	0.09 - 0.6	0.01
CB	SS	0.23	0.11 - 0.85	0.11
CF	HM	0.01	0.003 - 0.05	<.0001
CF	SS	0.01	0.004 0.06	<.0001
HM	SS	0.56	0.50 - 3.18	0.96

Multiple pairwise comparisons identified significant differences associated with each antibiotic resistance occurring within the various segments. CF – cattle feces (n = 382); CB – catch basin (n=137); SS – surrounding streams (n = 59); HM – clinical humans (n = 25) and municipal sewage treatment (n = 98) and BP** – beef processing plant (n = 4 **not included in this analyses) isolates.

Table S1b. Evaluation of differences in ampicillin resistance occurring among extended-spectrum cephalosporin resistant *E. coli* originating from multiple segments of the One Health continuum

Sample	Variable	Odds ratio	95% CI	P-value
CB	CF	0.99	22.19 - 388.89	<.0001
CB	HM	1.0	38 - >999.999	<.0001
CB	SS	0.46	0.32 - 2.19	0.98
CF	HM	0.84	0.59 - 47.76	0.41
CF	SS	0.01	0.002 - 0.04	<.0001
HM	SS	0.002	<0.001 - 0.02	<.0001

Table S1c. Evaluation of differences in streptomycin resistance occurring among extended-spectrum cephalosporin resistant *E. coli* originating from multiple segments of the One Health continuum

Sample	Variable	Odds ratio	95% CI	P-value
CB	CF	0.91	4.13 - 24.20	<.0001
CB	HM	0.47	0.43 - 1.74	0.98
CB	SS	0.39	0.30 - 1.43	0.69
CF	HM	0.08	0.04 - 0.19	<.0001
CF	SS	0.06	0.03 - 0.17	<.0001
HM	SS	0.43	0.34 - 1.64	0.88

Table S1d. Evaluation of differences in amoxicillin/clavulanate resistance occurring among extended-spectrum cephalosporin resistant *E. coli* originating from multiple segments of the One Health continuum

Sample	Variable	Odds ratio	95% CI	P-value
CB	CF	0.93	5.21 - 30.35	<.0001
CB	HM	0.73	1.24 - 5.92	0.06
CB	SS	0.31	0.19 - 1.04	0.22
CF	HM	0.18	0.11 - 0.41	<.0001
CF	SS	0.03	0.01 - 0.11	<.0001
HM	SS	0.14	0.06 - 0.44	0.002

Table S1e. Evaluation of differences in ceftiofur resistance occurring among extended-spectrum cephalosporin resistant *E. coli* originating from multiple segments of the One Health continuum

Sample	Variable	Odds ratio	95% CI	P-value
CB	CF	0.99	11.98 - 635.19	<.0001
CB	HM	1.00	46.4 - >999.99	<.0001
CB	SS	0.53	0.35 - 3.54	1.00
CF	HM	0.89	2.24 - 31.57	0.01
CF	SS	0.01	0.002 - 0.09	<.0001
HM	SS	0.002	<0.001 - 0.02	<.0001

**Ceftiofur was not modeled in generic *E. coli* due to low resistance prevalence and consequent inability for the model to converge. Therefore, ceftazidime is not presented.

Table S1f. Evaluation of differences in ceftazidime resistance occurring among extended-spectrum cephalosporin resistant *E. coli* originating from multiple segments of the One Health continuum

Sample	Variable	Odds ratio	95% CI	P-value
CB	CF	0.96	8.05 - 81.98	<.0001
CB	HM	0.86	2.26 - 16.44	0.002
CB	SS	0.40	0.27 - 1.58	0.77
CF	HM	0.19	0.12 - 0.49	0.0005
CF	SS	0.02	0.01 - 0.1	<.0001
HM	SS	0.10	0.03 - 0.34	0.0008

**Ceftazidime was not modeled in generic *E. coli* due to low resistance prevalence and consequent inability for the model to converge

Table S1g. Evaluation of differences in sulfisoxazole resistance occurring among extended-spectrum cephalosporin resistant *E. coli* originating from multiple segments of the One Health continuum

Sample	Variable	Odds ratio	95% CI	P-value
CB	CF	0.95	6.89 - 52.82	<.0001
CB	HM	0.66	0.90 - 4.05	0.32
CB	SS	0.52	0.49 - 2.45	1.00
CF	HM	0.09	0.043 - 0.23	<.0001
CF	SS	0.05	0.02 - 0.16	<.0001
HM	SS	0.37	0.25 - 1.32	0.54

Table S1h. Evaluation of differences in florfenicol resistance occurring among extended-spectrum cephalosporin resistant *E. coli* originating from multiple segments of the One Health continuum

Sample	Variable	Odds ratio	95% CI	P-value
CB	CF	0.88	3.16 - 16.24	<.0001
CB	HM	0.09	0.04 - 0.29	0.0002
CB	SS	0.35	0.24 - 1.20	0.42
CF	HM	0.01	0.004 - 0.05	<.0001
CF	SS	0.07	0.03 - 0.22	<.0001
HM	SS	0.84	1.96 - 14.10	0.006

Table S1i. Evaluation of differences in trimethoprim-sulfamethoxazole resistance occurring among extended-spectrum cephalosporin resistant *E. coli* originating from multiple segments of the One Health continuum

Sample	Variable	Odds ratio	95% CI	P-value
CB	CF	0.87	2.99 - 14.28	<.0001
CB	HM	0.84	2.17 - 11.88	0.001
CB	SS	0.48	0.41 - 2.15	0.1
CF	HM	0.44	0.46 - 1.31	0.77
CF	SS	0.13	0.06 - 0.35	0.0002
HM	SS	0.16	0.072 - 0.48	0.003

**Trimethoprim/sulfamethoxazole was not modeled for generic *E. coli* due to low resistance prevalence and consequent inability for the model to converge.

Table S1j. Evaluation of differences in oxytetracycline resistance occurring among generic *E. coli* originating from multiple segments of the One Health continuum

Sample	Variable	Odds ratio	95% CI	P-value
CB	**CF**	0.93	5.75 - 28.67	<.0001
CB	BP	0.97	11.72 - 122.98	<.0001
CB	MS	0.35	0.26 - 1.14	0.43
CB	SS	0.79	1.82 - 7.59	0.004
CF	BP	0.75	1.44 - 6.08	0.03
CF	MS	0.04	0.02 - 0.12	<.0001
CF	SS	0.22	0.16 - 0.53	0.001
BP	MS	0.01	0.004 - 0.06	<.0001
BP	SS	0.09	0.04 - 0.24	<.0001
MS	SS	0.87	2.74 - 17.21	0.001

In the generic *E. coli* sub-population, MS isolates served as human-sourced isolates for statistical analyses. CF – cattle feces (n = 142); CB – catch basin (n=185); SS – surrounding streams (n = 81); municipal sewage treatment (n = 96) and BP – beef processing plant (n = 159).

Table S1k. Evaluation of differences in ampicillin resistance occurring among generic *E. coli* originating from multiple segments of the One Health continuum

Sample	Variable	Odds ratio	95% CI	P-value
CB	**CF**	0.16	0.09 - 0.37	<.0001
CB	BP	0.13	0.07 - 0.32	<.0001
CB	MS	0.24	0.16 - 0.67	0.01
CB	SS	0.08	0.03 - 0.24	<.0001
CF	BP	0.45	0.43 - 1.59	0.98
CF	MS	0.64	0.88 - 3.45	0.47
CF	SS	0.32	0.2 - 1.17	0.46
BP	MS	0.68	1.03 - 4.35	0.23
BP	SS	0.37	0.23 - 1.47	0.76
MS	SS	0.22	0.11 - 0.71	0.06

Table S1l. Evaluation of differences in amoxicillin/clavulanate resistance occurring among generic *E. coli* originating from multiple segments of the One Health continuum

Sample	Variable	Odds ratio	95% CI	P-value
CB	**CF**	0.08	0.03 - 0.31	0.001
CB	BP	0.10	0.04 - 0.38	0.004
CB	MS	0.19	0.08 - 0.71	0.08
CB	SS	0.03	0.004 - 0.31	0.02

CF	BP	0.57	0.4 - 4.57	0.99
CF	MS	0.73	0.84 - 8.73	0.44
CF	SS	0.28	0.04 - 3.51	0.91
BP	MS	0.67	0.63 - 6.42	0.75
BP	SS	0.22	0.03 - 2.61	0.79
MS	SS	0.12	0.02 - 1.26	0.39

Table S1m. Evaluation of differences in streptomycin resistance occurring among generic *E. coli* originating from multiple segments of the One Health continuum

Sample	Variable	Odds ratio	95% CI	P-value
CB	**CF**	0.81	2.10 - 8.87	0.00
CB	BP	0.89	3.73 - 18.98	<.0001
CB	MS	0.53	0.46 - 2.69	1.00
CB	SS	0.75	1.28 - 7.06	0.09
CF	BP	0.66	1.13 - 3.36	0.11
CF	MS	0.20	0.11 - 0.58	0.01
CF	SS	0.41	0.35 - 1.37	0.81
BP	MS	0.12	0.05 - 0.32	0.00
BP	SS	0.26	0.18 - 0.72	0.04
MS	SS	0.73	1.06 - 6.96	0.22

Table S1n. Evaluation of differences in sulfisoxazole resistance occurring among generic *E. coli* originating from multiple segments of the One Health continuum

Sample	Variable	Odds ratio	95% CI	P-value
CB	CF	0.85	2.07 - 14.67	0.01
CB	BP	0.91	3.34 - 27.45	0.0005
CB	MS	0.71	0.78 - 7.57	0.52
CB	SS	0.78	1.11 - 11.27	0.20
CF	CF	0.63	0.87 - 3.45	0.49
CF	CF	0.31	0.18 - 1.09	0.38
CF	CF	0.39	0.26 - 1.57	0.85
BP	BP	0.20	0.10 - 0.66	0.04
BP	BP	0.27	0.15 - 0.93	0.20
MS	SS	0.59	2.07 - 14.67	0.96

Table S1o. Evaluation of differences in florfenicol resistance occurring among generic *E. coli* originating from multiple segments of the One Health continuum

Sample	Variable	Odds ratio	95% CI	P-value
CB	CF	0.92	0.55 - 4.47	0.05
CB	BP	0.94	0.01 - 0.59	0.03
CB	MS	0.43	0.01 - 0.82	1.00
CB	SS	0.92	0.44 - 3.84	0.09
CF	CF	0.57	0.01 - 0.40	0.97
CF	CF	0.06	0.00 - 0.55	0.15
CF	CF	0.50	0.30 - 2.31	1.00
BP	BP	0.05	0.05 - 10.81	0.10
BP	BP	0.43	2.02 - 97.54	0.98
MS	SS	0.94	1.47 - 240.67	0.18

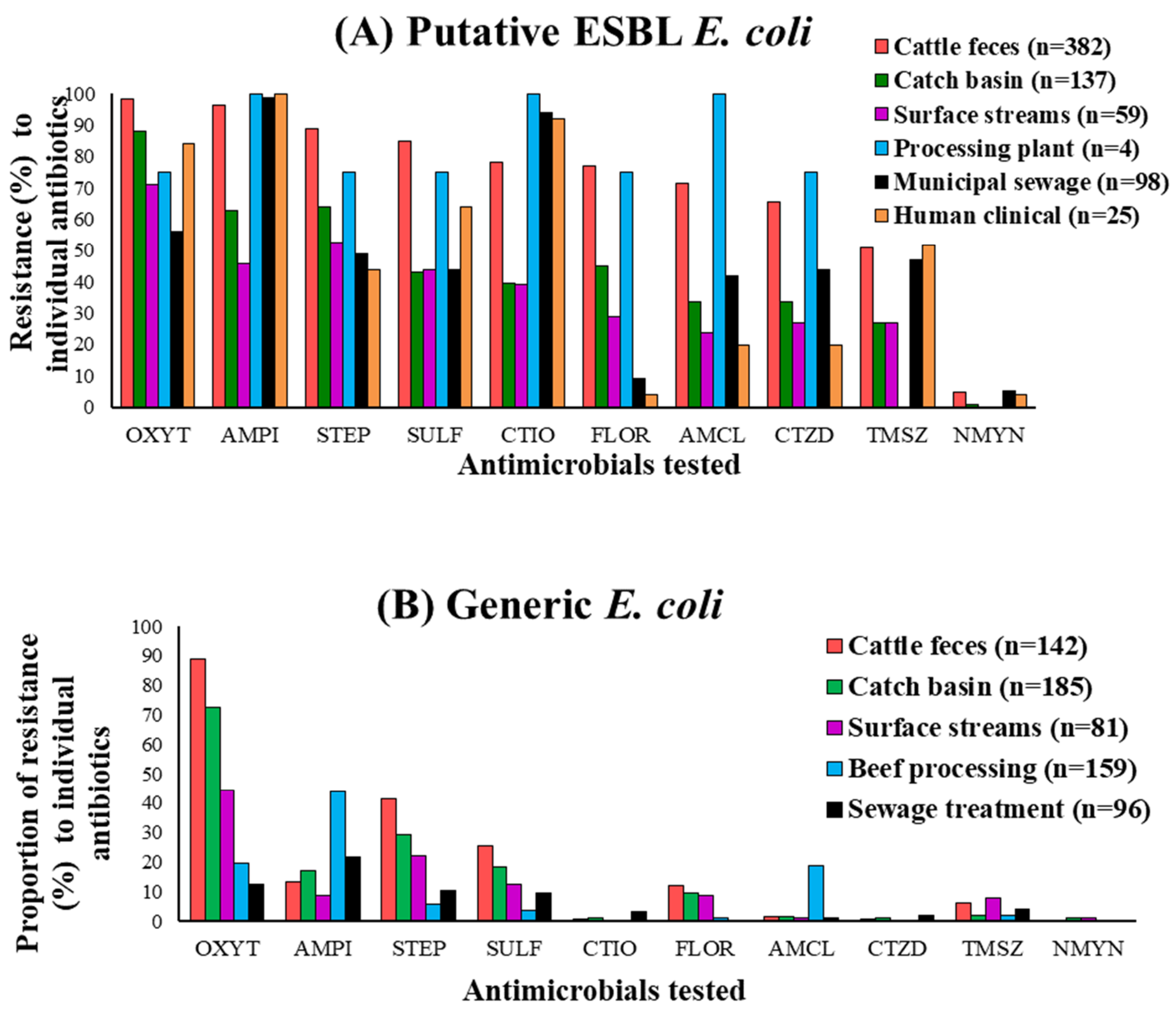

Figure S1: Trends of antimicrobial resistance prevalence in (A) extended-spectrum cephalosporin-resistant *E. coli* and (B) generic *E. coli* populations with specificity to sample origin, expressed as percentages. In (A), *E. coli* resistance to individual antibiotics differ across sources (P<0.001), where as in (B) *E. coli* did not differ across sources (P < 0.99), although differences were observed per source*antibiotic interaction in both populations (P<0.001).

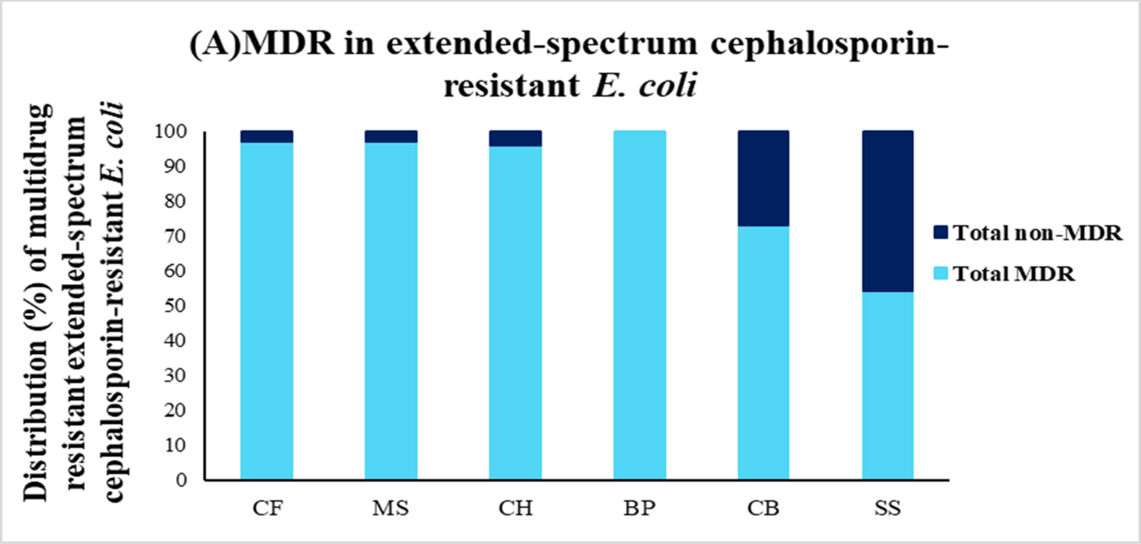

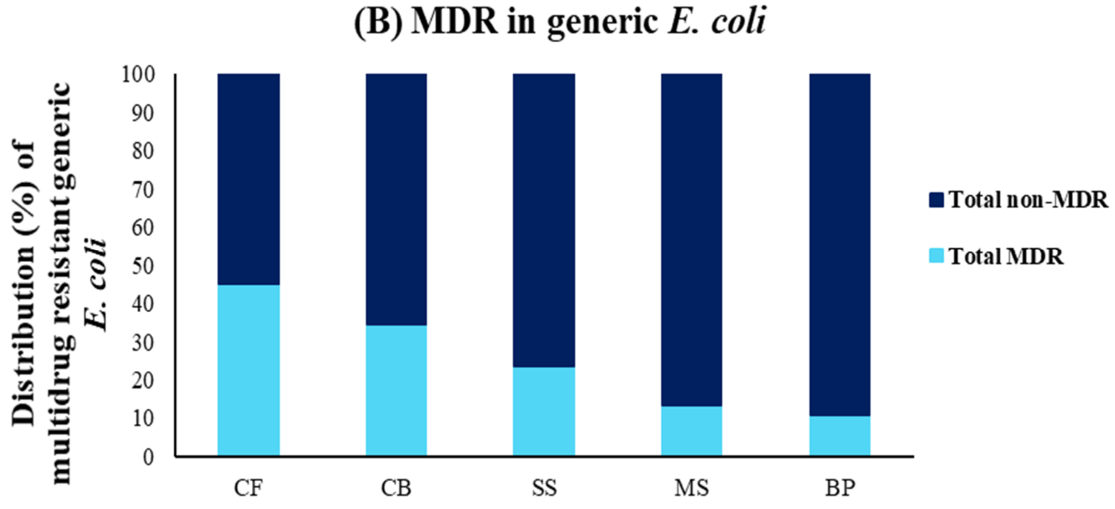

Figure S2: Prevalence of total multidrug resistance in (resistance to antibiotics in two classes and above in (A) extended-spectrum cephalosporin-resistantand (B) generic *E. coli* from CF – cattle feces, MS – municipal Sewage, CH – clinical ill humans, BP – beef processing, CB – catch basin, SS – surface streams

Table S2: Patterns of antimicrobial resistance in extended-spectrum cephalosporin-resistant *E. coli* showing resistance to six or more tested agents.

Patterns	Number of antibiotics	Number of isolates (prevalence)	Prevalence (%)
AMPI–CTZD–AMCL–CTIO–STEP–SULF–FLOR–OXYT–TMSZ–NMYN	10	5	1.7
AMPI–CTZD–AMCL–CTIO–STEP–SULF–FLOR–OXYT–TMSZ	9	123	41.3
AMPI–CTZD–CTIO–STEP–SULF–FLOR–OXYT–TMSZ–NMYN	9	4	1.3
AMPI–CMCL–CTZD–CTIO–STEP–SULF–FLOR–OXYT	8	105	35.2
AMPI–AMCL–CTZD–CTIO–STEP–SULF–OXYT	8	4	1.3
AMPI–AMCL–CTZD–CTIO–STEP–FLOR–OXYT	8	1	0.3
AMPI–AMCL–CTZD–STEP–SULF–FLOR–OXYT–TMSZ	8	1	0.3
AMPI–CTIO–STEP–SULF–FLOR–OXYT–TMSZ–NMYN	7	1	0.3
AMPI–CTIO–AMCL–STEP–SULF–OXYT–TMSZ–NMYN	8	5	1.7
AMPI–AMCL–CTIO–STEP–SULF–OXYT	7	3	1.0
AMPI–AMCL–CTZD–STEP–SULF–FLOR–OXYT	7	9	3.0
AMPI–AMCL–CTZD–STEP–FLOR–OXYT–TMSZ	7	1	0.3
AMPI–CTZD–CTIO–STEP–SULF–OXYT–TMSZ	7	5	1.7
AMPI–AMCL–CTZD–STEP–SULF–FLOR–OXYT	8	2	0.7
AMPI–CTIO–STEP–SULF–OXYT–TMSZ–NMYN	7	2	0.7
AMPI–CTIO–STEP–SULF–OXYT–FLOR–NMYN	7	1	0.3
AMPI–CTIO–STEP–SULF–OXYT–FLOR–TMSZ	7	9	3.0
AMPI–CTIO–STEP–SULF–OXYT–TMSZ	6	16	5.4
AMPI–CTIO–CTZD–OXYT–FLOR–TMSZ	6	1	0.3

Ampicillin - AMPI, amoxicillin/clavulanic acid - AMCL, ceftiofor (ceftiofur) - CTIO, ceftazadime (ceftazidime) - CTZD, streptomycin - STEP, neomycin - NMYN, oxytetracycline - OXYT, florfenicol - FLOR, trimethoprim/sulfamethoxazole - TMSZ, sulfisoxazole – SULF.

Table S3: Multidrug and ESBL phenotype versus β-lactamase and AmpC resistance genotypes in extended-spectrum cephalosporin-resistant *E. coli* isolated from different sources

Gene/Source	CHumans	MSewage	CFeces	CBasins	SStreams	BProcessing
ESBL phenotype	64	48	22.5	10.9	15.3	0
MDR phenotype	96	96.9	97.1	73	54.2	100
Total β-lactamase gene occurrence	100	80.6	35.3	27	22	0
SHV	0	3.1	1.8	0	0	0
TEM	48	34.7	15.4	19.7	15.3	0
OXA	28	14.3	0	0	0	0
CTXM	96	67.3	25.4	11.7	15.3	0
CTXM-1	72	33.7	18.6	11.7	15.3	0
CTX-M 2	24	19.4	5.2	0	0	0
CTX-M 9	24	28.6	5.8	0	0	0
CMY	32	50	87.2	91.2	93.2	50
